# Dendritic Cell Targeting of Bovine Viral Diarrhea Virus E2 Protein Expressed by *Lactobacillus casei* Effectively Induces Antigen-Specific Immune Responses via Oral Vaccination

**DOI:** 10.3390/v11060575

**Published:** 2019-06-25

**Authors:** Yixin Wang, Baohua Feng, Chao Niu, Shuo Jia, Chao Sun, Zhuo Wang, Yanping Jiang, Wen Cui, Li Wang, Yigang Xu

**Affiliations:** 1 Heilongjiang Key Laboratory for Animal Disease Control and Pharmaceutical Development, College of Veterinary Medicine, Northeast Agricultural University, Harbin 150030, China; JCwangyixin@163.com (Y.W.); fengbaohua66@163.com (B.F.); niuchao19931002@163.com (C.N.); jiashuo0508@163.com (S.J.); 18345189115@139.com (C.S.); wangzhuo0108@163.com (Z.W.); jiangyanping8198@163.com (Y.J.); cuiwen_200@163.com (W.C.); 2 Northeastern Science Inspection Station, China Ministry of Agriculture Key Laboratory of Animal Pathogen Biology, Harbin 150030, China

**Keywords:** bovine viral diarrhea virus (BVDV), glycoprotein E2, probiotics vaccine, dendritic cell-targeting delivery, oral immunization

## Abstract

Bovine viral diarrhea caused by bovine viral diarrhea virus (BVDV) is an important disease in cattle, resulting in significant economic losses to the cattle industry worldwide. In order to develop an effective vaccine against BVDV infection, we constructed a dendritic cell (DC)-targeting oral probiotic vaccine (pPG-E2-DCpep/LC W56) using *Lactobacillus casei* as antigen delivery carrier to express BVDV glycoprotein E2 fused with DC-targeting peptide, and the immunogenicity of orally administered probiotic vaccine was evaluated in mice model. Our results showed that after immunization with the probiotic vaccine, significantly levels of antigen-specific sera IgG and mucosal sIgA antibodies (*p* < 0.05) with BVDV-neutralizing activity were induced in vivo. Challenge experiment showed that pPG-E2-DCpep/LC W56 can provide effective immune protection against BVDV, and BVDV could be effectively cleared from the intestine of immunized mice post-challenge. Moreover, the pPG-E2-DCpep/LC W56 could efficiently activate DCs in the intestinal Peyer’s patches, and significantly levels of lymphoproliferative responses, Th1-associated IFN-γ, and Th2-associated IL-4 were observed in mice immunized with pPG-E2-DCpep/LC W56 (*p* < 0.01). Our results clearly demonstrate that the probiotic vaccine could efficiently induce anti-BVDV mucosal, humoral, and cellular immune responses via oral immunization, indicating a promising strategy for the development of oral vaccine against BVDV.

## 1. Introduction

Bovine viral diarrhea virus (BVDV) is the causative agent of bovine viral diarrhea (BVD), which is an economically important viral disease in cattle that is endemic in many countries worldwide, causing considerable economic losses in the global dairy/cattle industry [1,2,3,4,5]. BVDV is an enveloped, single-stranded RNA virus that belongs to the genus *Pestivirus* within the family *Flaviviridae* [6], and based on the nucleotide sequence of its 5’ untranslated region, BVDV is divided into two distinct genotypes, BVDV-1 and BVDV-2, with cytopathic (CP) and non-cytopathic (NCP) biotypes for each genotype [7,8,9]. BVDV infection in cattle can cause respiratory disease, diarrhea, mucosal disease syndrome, weak calf syndrome, and abortion [10]. Currently, vaccines for BVD are available, including inactivated vaccines and live attenuated vaccines, but, in some cases, the efficacy of such attenuated or killed BVDV vaccines under controlled experimental conditions and under field conditions has also been controversial [11], e.g. modified live CP-BVDV vaccines can induce severe mucosal disease in persistently infected (PI) calves. Moreover, the combination of identification and removal of PI calves, the implementation of appropriate biosecurity measures, on-going surveillance and eradication is recognized to be a successful strategy to control BVDV infection [11,12,13,14]. However, it inevitably requires tremendous financial support [15,16]. Therefore, it is necessary to develop effective vaccines against BVDV infection.

Naturally, BVDV infection often initiates at mucosal surfaces, including nasal [17] and intestinal mucosa tissues [18]. Therefore, the design of a novel vaccine that can efficiently induce secretory immunoglobulin A (sIgA)-based protective mucosal immunity and further trigger IgG-based protective systemic immune responses could effectively prevent BVDV from invading the body via the mucosa and further spreading to the systemic circulation. Recently, there has been an increase in interest in using probiotics as antigen delivery carriers to develop oral mucosal vaccines against enteric viruses, particularly lactic acid bacteria, and their potential to deliver vaccine antigens to the intestinal mucosal system to elicit protective immune responses has been investigated during the last decade [19,20,21,22,23,24]. In addition, the intestinal mucosal dendritic cell (DC)-targeting oral vaccine has been suggested as a promising strategy for improving the delivery efficiency of vaccine antigens to the mucosal immune system by oral administration to further elicit effective mucosal immune responses against infection [25,26,27]. Studies have confirmed that the intestinal DC-targeting of genetically engineered probiotic lactobacillus vaccine could elicit antigen-specific mucosal and systemic immune responses against pathogen infection via oral vaccination [23,27], exhibiting a better immunogenicity.

Moreover, the induction of neutralizing antibodies is crucial to develop an effective vaccine against BVDV infection. Studies have shown that the major glycoprotein E2 of BVDV encompasses major antigenic domains with the capacity to induce neutralizing antibodies, which has therefore been studied extensively as a potential candidate for the development of vaccines against BVDV [28,29,30,31]. In the present study, *Lactobacillus casei* was used as an antigen delivery vehicle, and a novel approach involving DC-targeting of oral probiotic vaccine constitutively expressing BVDV envelope glycoprotein E2 was developed. Its immunogenicity in mice to induce protective mucosal and systemic immune responses against BVDV infection was also evaluated via oral vaccination.

## 2. Materials and Methods

Animal experiments were carried out in accordance with the international (OIE Terrestrial animal health code) and national guidelines (CNAS-CL06:2018) for the care and use of laboratory animals. The project 2017NEAU09315 was approved by the Committee on the Ethics of Animal Experiments of Northeast Agricultural University of China (29 Sep 2017). 

### 2.1. Bacterial Strain, Virus, Plasmid, and Animals

*Lactobacillus casei* strain W56 (LC W56) isolated from the cattle feces by our lab was cultured anaerobically in de Man, Rogosa, and Sharpe (MRS) broth (Sigma, St. Louis, MO, USA) at 37 °C. Cytopathic (CP) BVDV-1 strain ZD-2018 was isolated from Heilongjiang Province, China, and was propagated in MDBK cells at 37 °C under 5% CO_2_, which was purified by plaque method prior to experimental work being performed. A constitutive expression plasmid pPG-T7g10 used for developing recombinant lactobacillus was constructed in our laboratory [24], which contained a T7g10 transcriptional enhancer, an HCE strong promoter obtained from the D-amino acid aminotransferase, a PgsA anchor obtained from *Bacillus subtilis*, and an rrnBT1T2 terminator. Five-week-old specific pathogen-free (SPF) BALB/c mice were obtained from Changsheng Biotechnology Company (Shenyang, China), and kept under SPF conditions with free access to standard diet and water.

### 2.2. Construction of Recombinant Lactobacillus

**Figure 1:**
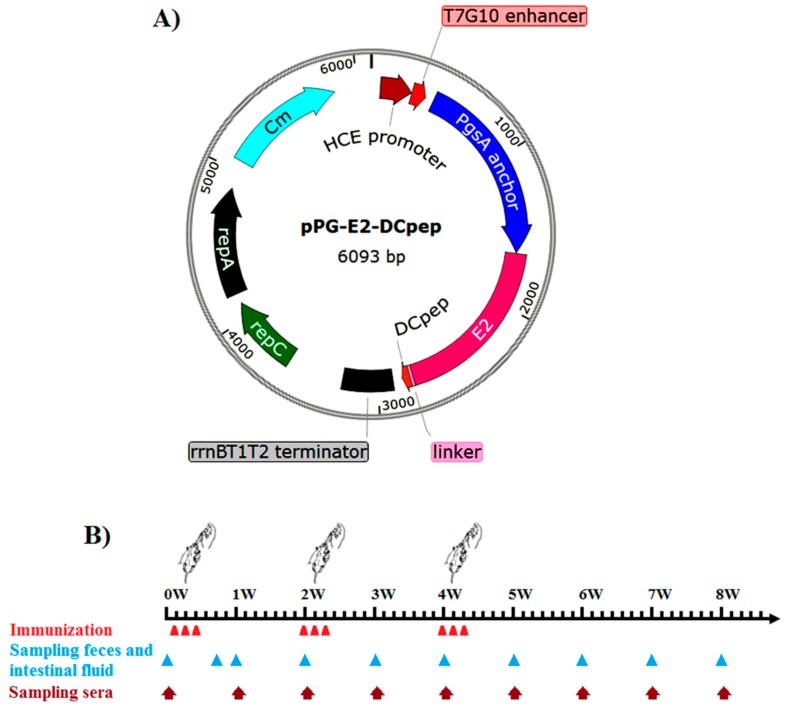
Schematic diagram of the recombinant plasmid pPG-E2-DCpep (**A**), and the immunization protocol and sampling schedule after the primary immunization (**B**).

The genomic RNA of the BVDV strain ZD-2018 propagated in MDBK cells was extracted using a Trizol Total RNA Isolation Kit (Invitrogen, Grand Island, NY, USA) according to the manufacturer’s instructions, and then, the first-strand cDNA was reverse transcribed using a QuantiTect Reverse Transcription Kit (QIAGEN, Hilden, Germany), according to the manufacturer’s instructions. Subsequently, the gene encoding BVDV glycoprotein E2 was amplified by polymerase chain reaction (PCR) with BVDV-E2-F/R primers (Table 1) using the cDNA as template, and subcloned into pMD-19T (Takara, Dalian, China) followed by sequencing, giving rise to recombinant plasmid pMD-E2. Then, the fusion gene *E2* and DC-targeting peptide (DCpep) gene was produced by PCR with BVDV-E2-F/E2-DCpep primers (Table 1) using the plasmid pMD-E2 DNA as template, and subcloned as a *Sac* I/*Apa* I gene fragment into the plasmid pPG-T7g10, giving rise to recombinant plasmid pPG-E2-DCpep (Figure 1A), where the genes encoding E2 and DCpep were linked together by oligonucleotides encoding a flexible linker (Table 1). After that, the plasmid pPG-E2-DCpep was electroporated into LC W56 competent cells followed by validation by PCR and sequencing, generating the recombinant lactobacillus pPG-E2-DCpep/LC W56. Simultaneously, a recombinant lactobacillus pPG-E2/LC W56 was constructed as control.

### 2.3. Expression and Identification of the Fusion Protein

The recombinant lactobacilli (pPG-E2-DCpep/LC W56 and pPG-E2/LC W56) were grown overnight in MRS broth without the presence of any specific inducers at 37 °C, and were then centrifuged at 10,000× *g* for 5 min. The cell pellets were treated with 2× sodium dodecyl sulfate (SDS) loading buffer and lysed in boiling water bath for 10 min. After centrifugation at 12,000× *g* for 10 min, the bacterial proteins in supernatant were separated by 12% SDS-polyacrylamide gel electrophoresis (SDS-PAGE), and electrotransferred onto a polyvinylidene fluoride (PVDF) membrane (Roche, Nutley, NJ, USA). Subsequently, the membranes were incubated with mouse anti-E2 monoclonal antibody (1:1000) prepared in our lab as the primary antibody, and horseradish peroxidase (HRP)-conjugated goat anti-mouse IgG antibody (1:2000) (Sigma) as the secondary antibody. The results were visualized using a chemiluminescent substrate reagent (Thermo Fisher Scientific, San Jose, CA, USA), according to the manufacturer’s instructions. Moreover, an indirect immunofluorescence assay was performed to identify the cell surface display of the fusion protein by the pPG-E2-DCpep/LC W56. Briefly, the recombinant lactobacilli (pPG-E2-DCpep/LC W56 and pPG-E2/LC W56) were grown in MRS broth at 37 °C for 12 h, and after washing twice with sterile PBS and centrifuging at 12,000× *g* for 10 min, the cells were incubated with mouse anti-E2 monoclonal antibody (1:200) and fluorescein isothiocyanate (FITC)-labeled goat anti-mouse IgG antibody (1:1000) (Thermo Fisher Scientific). After washing thrice with sterile PBS and centrifuging, the cell pellets were resuspended in sterile PBS and laid on a glass slide, and then, fluorescence reaction on the cell surface of pPG-E2-DCpep/LC W56 and pPG-E2/LC W56 was observed by confocal microscopy, using pPG/LC W56 and LC W56 as negative control.

### 2.4. Determination of Adherence Ability of Recombinant Lactobacillus in Intestinal Tracts

The recombinant lactobacillus pPG-E2-DCpep/LC W56 was cultured overnight in MRS broth at 37 °C and were centrifuged at 12,000× *g* for 5 min. After washing twice with sterile PBS, the cells were adjusted to a concentration of 10^10^ CFU/mL followed by labeling with 5’-(and 6’)-carboxyfluorescein diacetate succinimidyl ester, cFDA-SE (Sigma) at 37 °C for 25 min. Before oral administration, the labeling efficiency of recombinant lactobacillus with cFDA-SE was analyzed by flow cytometry. After that, one group of 40 mice was orally dosed with 200 μL of approximately 10^10^ CFU/mL of cFDA-SE-labeled pPG-E2-DCpep/LC W56 for each mouse, and another group of 40 mice was fed with sterile PBS as control. On days 1, 3, 5, 7, 9, 11, 13, and 15 after oral administration, five mice from each group were selected randomly, and the duodenum, jejunum, ileum, and colon were extracted from each mouse and cut longitudinally followed by removal of any residual food particles or fecal material. Subsequently, after adding 200 μL of PBS to every 1.0 cm of tissue and dislodging microbes from intestinal mucosal surface, the presence of adhering cFDA-SE-labeled pPG-E2-DCpep/LC W56 in each intestinal tract was determined by flow cytometry.

### 2.5. Oral Immunization and Immune Sample Collection

The bacterial strains pPG-E2-DCpep/LC W56, pPG-E2/LC W56, and LC W56 were cultured overnight in MRS broth at 37 °C, and after washing twice with PBS, the cells were resuspended in PBS buffer supplemented with 5% casein peptone and 0.5% glucose to a final concentration of 10^10^ CFU/mL. A total of two hundred five-week-old SPF BALB/c mice were randomly divided into four groups (fifty mice for each group): pPG-E2-DCpep/LC W56 group, pPG-E2/LC W56 group, LC W56 group, and PBS group. All mice in pPG-E2-DCpep/LC W56 group, pPG-E2/LC W56 group, and LC W56 group were orally vaccinated with 200 μL of 10^10^ CFU/mL of pPG-E2-DCpep/LC W56, pPG-E2/LC W56, and LC W56, respectively, and mice in the PBS group received an equivalent quantity of PBS buffer. The immunization protocol was performed as previously described [19]. In brief, primary immunization was carried out on three consecutive days (days 1, 2, and 3), and a booster immunization was given on days 15, 16, and 17, and a second booster immunization was given on days 28, 29, and 30 (Figure 1B). After vaccination, samples (sera, feces, and intestinal mucus) of five mice selected randomly from each group were collected at each time point according to the schematic diagram shown in Figure 1B, and stored at −80 °C until further use.

### 2.6. Activation of Dendritic Cells in Intestinal Peyer’s Patches by Recombinant Lactobacillus

To evaluate the potential effects of recombinant lactobacillus on DCs in intestinal Peyer’s patches (PPs), the PPs in the small intestine of mice of each group were isolated on day 5 after the primary immunization, and lymphocytes in the PPs were collected as per the method described previously [32,33] with some modifications. Briefly, PPs excised from the serosa side of small intestine were placed in a Petri dish containing 5 mL of precooled Hank’s Balanced Salt Solution (HBSS) followed by gentle grinding; the cell suspension was filtered with a 200 µm stainless steel mesh followed by centrifugation at 500× *g* for 10 min; the cell pellets were resuspended in 8 mL of HBSS, and were layered over 5 mL of 67% Percoll (Sigma) in a 15-mL conical centrifuge tube followed by centrifugation at 600× *g* for 20 min; the cells at the interface were harvested, washed twice with HBSS, and resuspended in HBSS to a final concentration of 10^6^/mL. Subsequently, the cells were incubated with anti-CD16/CD32 antibody (BD Biosciences, San Diego, CA, USA) to block Fc receptors, and after washing twice with HBSS, the cells were stained with FITC-conjugated anti-CD40, CD86 antibody (BD Biosciences) followed by flow cytometry analysis.

### 2.7. ELISA of Antibody Levels Induced by Rrecombinant Lactobacillus

For preparation of the immune samples, serum samples were collected and stored at −80 °C until further use; approximately 0.2 g of feces samples were dissolved in 500 μL of PBS containing 1 mmol/L phenylmethylsulfonyl fluoride and 1% bovine serum albumin (Sigma), mixed thoroughly, and incubated overnight with gentle shaking at 4 °C, and after centrifugation at 10,000× *g* for 5 min, the supernatants were stored at −80 °C until further use; intestinal mucus samples scraped from intestine tracts of the immunized mice in each group were mixed thoroughly with 500 μL of HEPES buffer (Thermo Fisher Scientific), and extracted with gentle shaking at 4 °C for 2 h followed by centrifugation; the supernatants were stored at −80 °C until further required. After that, the levels of antigen-specific IgG antibody in sera and intestinal mucosal sIgA antibody in feces/intestinal mucus collected from the mice from each group at each indicated time point after immunization were determined by ELISA on the same day, respectively. In brief, a 96-well polystyrene plate was coated overnight at 4 °C with BVDV propagated in MDBK cells followed by washing; the plate was blocked with 5% skimmed milk at 37 °C for 2 h followed by washing; sera samples (diluted at 1:100) and supernatants of intestinal mucus and feces samples (diluted at 1:10) as the primary antibody were added into the plate in quintuplicate and incubated at 37 °C for 1 h followed by washing; HRP-conjugated goat anti-mouse IgG or IgA antibody (diluted at 1:5000) (Sigma) was added to the plate as the secondary antibody and incubated at 37 °C for one hour followed by washing. Subsequently, color development was carried out using tetramethylbenzidine (QIAGEN) as a colorimetric substrate, and absorbance at 490 nm was measured. In parallel, the sera against BVDV E2 protein obtained from BALB/c mice that were immunized with the purified E2 protein expressed by *E. coli* was used as positive control, and BALB/c mice sera without immunization were used as negative control. There was no anti-E2 IgA antibody used as positive control in this study. When ELISA was carried out, the assay was considered effective only when the ratio of OD positive serum control to OD negative serum control was greater than 2.

### 2.8. Determination of Neutralization Ability of Antibodies

The sera IgG and mucosal sIgA antibodies obtained from the mice orally immunized with the recombinant lactobacillus (pPG-E2-DCpep/LC W56, pPG-E2/LC W56) on day 42 after the primary immunization were used to evaluate the BVDV-neutralizing activity. Briefly, 50 μL of serum IgG antibody/mucosal sIgA antibody sample was serially diluted two-fold in a 96-well cell culture plate, and was then mixed thoroughly with 50 μL of 200× 50% tissue culture infective dose (TCID_50_) BVDV propagated in MDBK cells followed by incubation at 37 °C for 1 h. The antibody-virus mixture was transferred to MDBK cell monolayers in another 96-well cell plate followed by incubation at 37 °C in a 5% CO_2_ incubator for 3 days, and cytopathic effects (CPE) were observed. Simultaneously, the antibody samples obtained from the LC W56 group and PBS group were used as control. Each sample was analyzed with five technical replicates and eight biological replicates.

### 2.9. Measurement of Lymphocyte Proliferation and Cytokine Levels

For determination of the lymphocyte proliferation, five mice from each group were euthanized on day 42 after the primary immunization, and the splenocytes of each mouse were prepared to a final concentration of 5 × 10^6^ cells/mL. Then, 100 μL of cell suspensions were added into a 96-well cell plate in eight duplicates containing RPMI 1640 medium supplemented with 10% fetal bovine serum, and incubated at 37 °C in a 5% CO_2_ incubator. After that, the cells were respectively restimulated with 0.5 μg/mL and 5 μg/mL of recombinant E2 protein for 3 days, followed by addition of 10 μL of thiazolyl blue tetrazolium bromide, MTT (Sigma) into each well, and incubation at 37 °C for another 4 h followed by evaluation with the CellTiter 96^®^ AQueous Non-Radioactive Cell Proliferation Assay (Promega, Fitchburg, WI, USA) according to the manufacturer’s instructions. Subsequently, the absorbance at 570 nm was measured for each well. Concanavalin A (ConA, 5 μg/mL, Sigma) and cell culture medium was used as a positive control and negative control, respectively. The stimulation index was calculated as follows: SI = OD570 (sample)/OD570 (blank control) [24]. At the same time, the levels of Th1-associated cytokine interferon-γ (IFN-γ) and Th2-associated cytokine interleukin-4 (IL-4) in the culture supernatants of the splenocytes restimulated with 5 μg/mL of recombinant E2 protein were determined by ELISA Kit (Abcam, Cambridge, MA, USA) according to the manufacturer’s instructions. Moreover, splenocytes (5 × 10^6^ cells/mL) obtained from the five mice of each group were stained with anti-CD4-FITC antibody and anti-CD8-PE antibody (Abcam) at 37 °C for 30 min, respectively, and then, flow cytometry was performed using a FACS Caliber flow cytometry (Becton Dickinson, Franklin Lakes, NJ, USA) to determine T cell subsets.

### 2.10. Challenge Experiment

In order to evaluate the protective effects of the recombinant lactobacillus constructed in this study via oral vaccination, a challenge experiment was carried out using mice as animal model. Briefly, four groups of mice (45 mice for each group) were orally vaccinated with pPG-E2-DCpep/LC W56, pPG-E2/LC W56, LC W56, or PBS, according to the immunization protocol described above, and on day 42 after the primary immunization, the mice in each group were orally challenged with 200 μL containing 10^5^ TCID_50_ BVDV propagated in MDBK cells. During the 15-day challenge period, feces samples of mice in each group were collected per day, which were used for the detection of virus excretion by RT-PCR assay. At the same time, the intestine, blood, lung, and spleen samples of mice in each group were collected per day to detect the viral loads by SYBR Green real-time RT-PCR assay. Moreover, on day 15 post-challenge, the mice in each group were euthanized, and the histopathological changes in the intestine were observed, and viral antigens in the intestinal mucosa were detected by immunohistochemistry (IHC) assay with the HRP-conjugated mouse anti-E2 monoclonal antibody prepared in our lab.

### 2.11. Statistical Analysis

In this study, GraphPad Prism V5.0 software (GraphPad Software, San Diego, CA, USA) was used to perform statistical analyses for all data. The results of adherence ability, sera IgG antibody and mucosal sIgA antibody levels, neutralization ability of antibodies, and cytokines levels are shown as means ± standard errors of five replicates per test in a single experiment repeated thrice. Tukey’s multiple comparison tests and one-way analysis of variance (ANOVA) were used to analyze the significance of difference between groups. Value of *p* < 0.05 (*) was considered as significant, and *p* < 0.01(**) was considered as highly significant.

## 3. Results

### 3.1. Identification of the Protein of Interest Expressed by the Recombinant Lactobacilli

**Figure 2:**
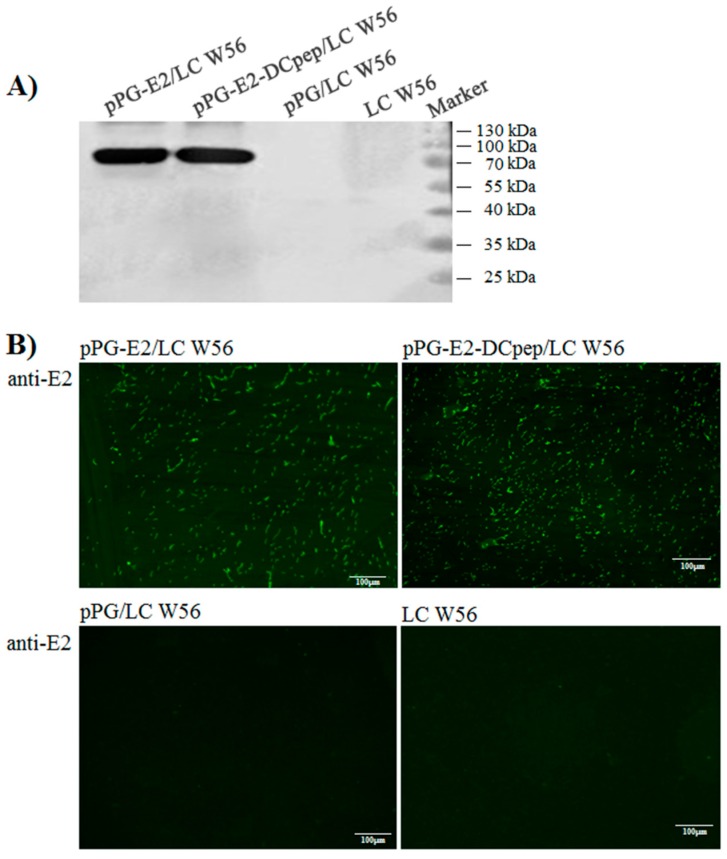
Identification of the protein of interest expressed by the recombinant strains using Western blot and immunofluorescence assay. As shown in (**A**), the cellular proteins of pPG-E2-DCpep/LC W56, pPG-E2/LC W56, pPG/LC W56, and LC W56 were separated by SDS-PAGE followed by identification by western blot with mouse anti-E2 monoclonal antibody, and specific immunoblot was respectively developed in the strains pPG-E2-DCpep/LC W56 and pPG-E2/LC W56, but not in pPG/LC W56 and LC W56. Moreover, as shown in (**B**), the results of immunofluorescence assay showed that green fluorescence was observed on the cell surface of pPG-E2-DCpep/LC W56 and pPG-E2/LC W56, but not on pPG/LC W56 and LC W56.

The cellular proteins of pPG-E2-DCpep/LC W56, pPG-E2/LC W56, pPG/LC W56, and LC W56 cultured overnight were identified by western blotting using mouse anti-E2 monoclonal antibody. Results showed a specific immunoblot band with expected size in pPG-E2-DCpep/LC W56 and pPG-E2/LC W56 (Figure 2A) respectively, but not in pPG/LC W56 and LC W56, indicating that the protein of interest can be constitutively expressed by the strains pPG-E2-DCpep/LC W56 and pPG-E2/LC W56. Moreover, the results of indirect immunofluorescence assay showed green fluorescence on the cell surface of the strains pPG-E2-DCpep/LC W56 and pPG-E2/LC W56 (Figure 2B), but not on the surface of pPG/LC W56 and LC W56, indicating that the protein of interest was displayed on the cell surface of the recombinant lactobacilli.

### 3.2. Colonization Ability of the Recombinant Lactobacillus in the Intestinal Tract

**Figure 3:**
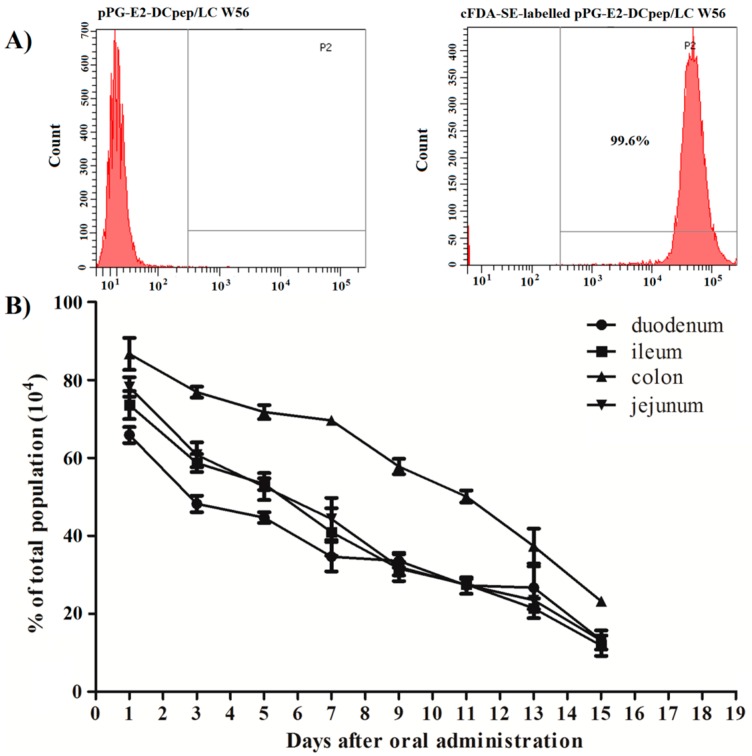
Colonization ability of the recombinant strain pPG-E2-DCpep/LC W56 in the intestinal tracts of mice. BALB/c mice were orally administrated with the cFDA-SE-labeled strain pPG-E2-DCpep/LC W56 (**A**) followed by extraction of the duodenum, jejunum, ileum, and colon of mice on days 1, 3, 5, 7, 9, 11, 13, and 15 after oral administration, and the microbes dislodged from mucosal surface were subjected to flow cytometric analysis. Results showed that pPG-E2-DCpep/LC W56 can adhere and colonize in the intestinal tracts of mice, and adhesion of the pPG-E2-DCpep/LC W56 was most prominent in the colon (**B**).

The mice were orally administrated with the recombinant lactobacillus pPG-E2-DCpep/LC W56 labeled with the cFDA-SE (labeling rate reached to 99.6% as shown in Figure 3A), and the duodenum, jejunum, ileum, and colon of mice were excised on days 1, 3, 5, 7, 9, 11, 13, and 15 after oral administration followed by dislodging of microbes from mucosal surface, and the colonization ability of the strain pPG-E2-DCpep/LC W56 in the intestinal tracts was analyzed by flow cytometry. As shown in Figure 3B, the pPG-E2-DCpep/LC W56 could adhere to and colonize in the intestinal tracts of mice, and from the first day, the detection rate of the cFDA-SE-labeled pPG-E2-DCpep/LC W56 gradually decreased each day. The adhesion of the pPG-E2-DCpep/LC W56 was most prominent in the colon.

### 3.3. Activation of DCs in PPs Stimulated by the Recombinant Lactobacillus

**Figure 4:**
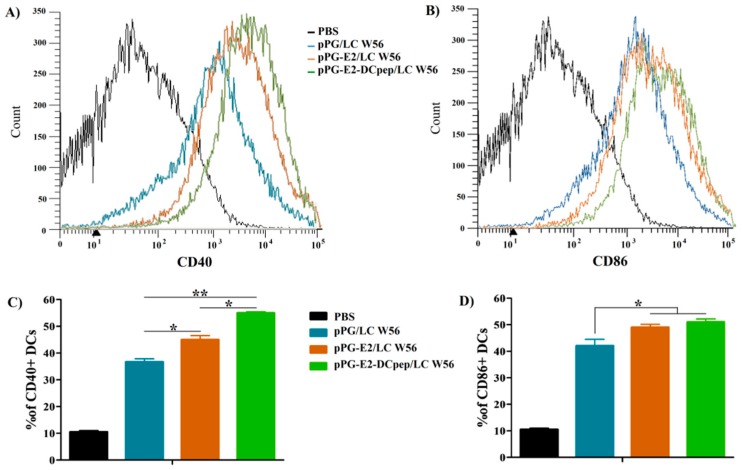
Activation of dendritic cells in intestinal Peyer’s patches stimulated by the recombinant strains. On day 5 after the primary immunization, DCs were isolated from the PPs in the small intestine of the mice orally administrated with pPG-E2-DCpep/LC W56, pPG-E2/LC W56, pPG/LC W56, and PBS, and the expression levels of costimulatory molecules of DCs, CD40 (**A**) and CD86 (**B**), were determined by flow cytometry, and the percentage of CD40+ DCs and CD86+ DCs was shown in panel (**C**) and (**D**), respectively.

On day 5 after the primary immunization, the levels of costimulatory molecules of DCs, CD40 and CD86, in the PPs excised from the small intestine of the mice in pPG-E2-DCpep/LC W56 group, pPG-E2/LC W56 group, pPG/LC W56 group, and PBS group were subjected to flow cytometric analysis. Compared to that in the PBS group, significant levels (*p* < 0.05) of CD40^+^ (Figure 4A) and CD86^+^ (Figure 4B) were induced by the strains pPG-E2-DCpep/LC W56, pPG-E2/LC W56, and pPG/LC W56. The level of CD40^+^ stimulated by pPG-E2-DCpep/LC W56 was significantly higher than those stimulated by pPG-E2/LC W56 (*p* < 0.05) and pPG/LC W56 (*p* < 0.01) (Figure 4C), and there was no difference (*p* > 0.05) in the level of CD86^+^ stimulated by pPG-E2-DCpep/LC W56 and pPG-E2/LC W56, respectively, but both showed significant difference to pPG/LC W56 group (Figure 4D). Moreover, the levels of costimulatory molecules stimulated by pPG-E2/LC W56 were significantly higher (*p* < 0.01) than those stimulated by pPG/LC W56, indicating that BVDV E2 protein could promote DCs maturation.

### 3.4. Antigen-Specific Serum IgG Antibody Levels Induced by Recombinant Lactobacillus

On days 0, 7, 14, 21, 28, 35, 42, 49, and 56 after the primary vaccination, the antigen-specific IgG antibody levels in sera obtained from the mice in each group were determined by indirect ELISA assay.

**Figure 5:**
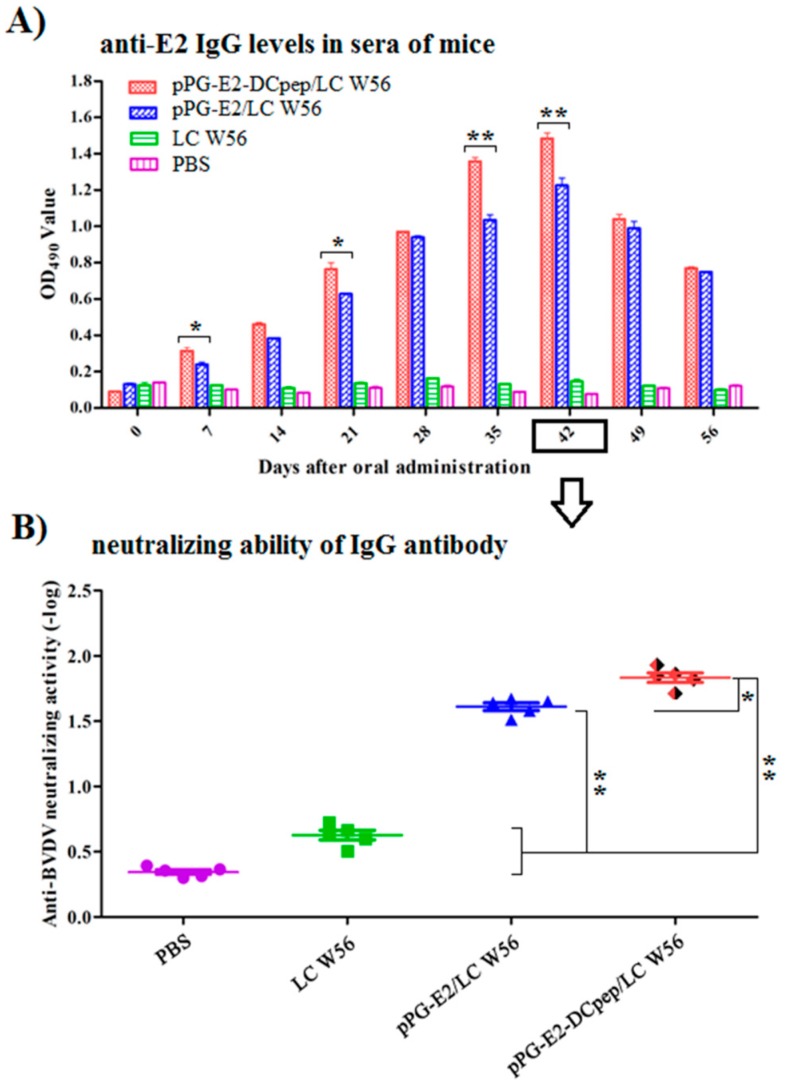
Determination of antigen-specific IgG antibody in sera of mice orally immunized with the recombinant strains at different time points after immunization and its BVDV-neutralizing activity. Using BVDV as the coating antigen, ELISA was used to determine the levels of anti-BVDV IgG antibody in serum samples (**A**) and the BVDV-neutralizing activity (**B**) of the serum antibody collected on days 42 after the primary immunization was determined. Data is represented as mean ± standard error value of each group. “*” means *p* < 0.05, “**” means *p* < 0.01.

As shown in Figure 5A, compared to that in the LC W56 and PBS groups, significant levels (*p* < 0.01) of specific IgG antibody were induced by the strains pPG-E2-DCpep/LC W56 and pPG-E2/LC W56 on the seventh day after the primary immunization. The levels of IgG antibody in sera induced by pPG-E2-DCpep/LC W56 were significantly different (*p* < 0.05) from those induced by pPG-E2/LC W56. Not surprisingly, no significant difference (*p* > 0.05) in the antigen-specific IgG antibody levels was observed between the LC W56 group and PBS group before and after immunization. 

Moreover, BVDV-neutralizing activity of the serum antibody collected from the mice in each group was determined on day 42 after the primary immunization (Figure 5B), and results showed that the serum antibody obtained from the mice orally immunized with pPG-E2-DCpep/LC W56 possessed a stronger BVDV-neutralizing activity (mean value of 1:64) compared to that in the pPG-E2/LC W56 group (*p* < 0.05), and LC W56 group and PBS group (*p* < 0.01).

### 3.5. Antigen-Specific Intestinal Mucosal sIgA Antibody Levels Induced by Recombinant Lactobacillus

**Figure 6:**
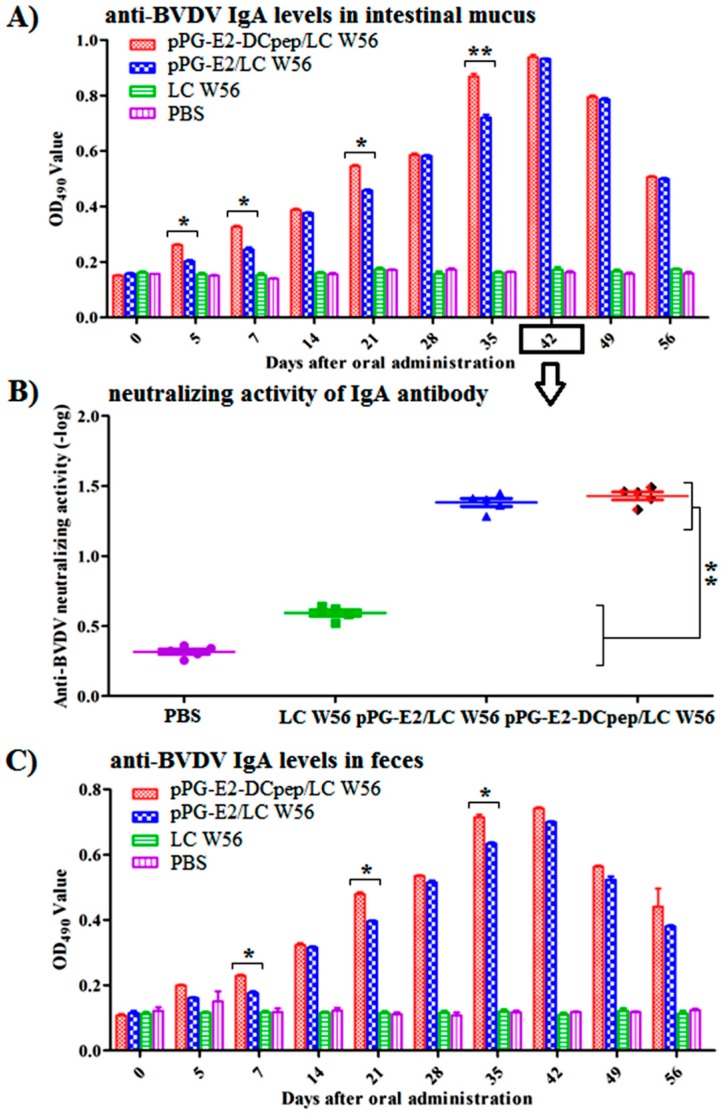
Determination of anti-BVDV mucosal sIgA antibody in intestinal mucus (**A**) and feces (**C**) samples collected from the immunized mice at different time points after immunization by ELISA using BVDV as the coating antigen, and BVDV-neutralizing activity of the intestinal mucosal sIgA antibody (**B**) collected on days 42 after the primary immunization. Data is represented as mean ± standard error value of each group. “*” means *p* < 0.05, “**” means *p* < 0.01.

As shown in Figure 6, on days 0, 5, 7, 14, 21, 28, 35, 42, 49, and 56 after the primary immunization, antigen-specific mucosal sIgA antibody levels were determined in intestinal mucus (Figure 6) and feces (Figure 6) samples obtained from the mice in each group. Results showed that from the 5th day after the primary immunization, a significantly higher level (*p* < 0.05) of mucosal sIgA antibody was detected in the mice orally immunized with pPG-E2-DCpep/LC W56 compared to that in the other groups. Moreover, the levels of mucosal sIgA antibody induced by pPG-E2/LC W56 were significantly different (*p* < 0.01) from those in the LC W56 group and PBS group after the first booster immunization, while no significant difference (*p* > 0.05) in mucosal sIgA antibody levels was observed between the LC W56 group and PBS group before and after immunization. Moreover, the BVDV-neutralizing activity of intestinal mucosal sIgA antibody collected on day 42 post-immunization from the mice in each group was determined (Figure 6B), and a stronger BVDV-neutralizing activity (mean value of 1:24) of mucosal sIgA antibody in the mice induced by pPG-E2-DCpep/LC W56 and pPG-E2/LC W56 was determined (*p* < 0.01) compared to that in the LC W56 group and PBS group.

### 3.6. CD4^+^ and CD8^+^ T Cell Detection

**Figure 7:**
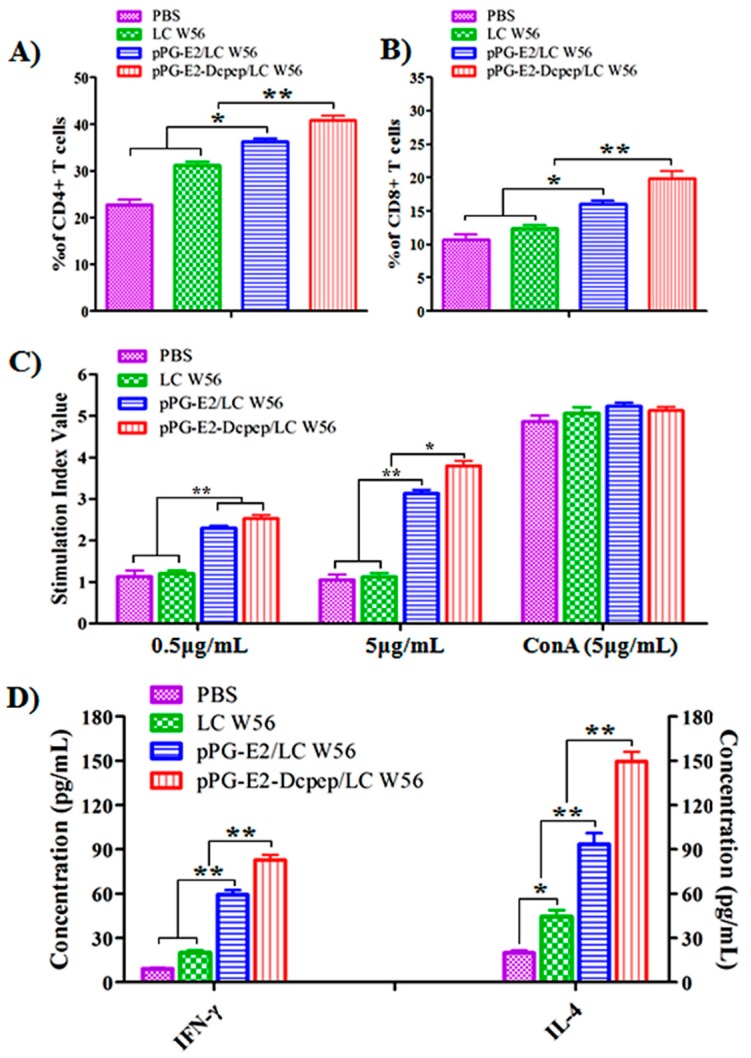
The percentage of CD4^+^ and CD8^+^ T cells determined in spleen lymphocytes of the mice orally immunized with the recombinant strains on days 42 after the primary immunization by flow cytometric analysis (**A**), and lymphoproliferation response after restimulation with the recombinant E2 protein determined by the MTT assay (**B**). The levels of Th1-associated cytokine IFN-γ and Th2-associated cytokine IL-4 produced by splenocytes post-restimulation (**C**,**D**). Data is represented as mean ± standard error value of each group. “*” means *p* < 0.05, “**” means *p* < 0.01.

On day 42 after the primary immunization, spleen lymphocytes of five mice selected randomly from each group were isolated, and the percentage of CD4^+^, and CD8^+^ T cells was analyzed by flow cytometry. Results showed that the percentages of CD4^+^ (Figure 7A), and CD8^+^ (Figure 7B) T cells in splenocytes of the mice immunized with the strain pPG-E2-DCpep/LC W56 were significantly higher (*p* < 0.01) compared to that in the pPG-E2/LC W56 group, and the percentages of CD4^+^, and CD8^+^ T cells in splenocytes of the mice in pPG-E2/LC W56 group were higher (*p* < 0.05) compared to that in the LC W56 group and PBS group. 

### 3.7. Lymphocyte Proliferation

The splenocytes of the five mice obtained from each group were restimulated by the recombinant E2 protein, and the lymphocyte proliferation response was detected by MTT assay. As shown in Figure 7C, the stimulation index with 0.5 μg/mL of recombinant E2 protein in the pPG-E2-DCpep/LC W56 group and pPG-E2/LC W56 group was significantly higher compared to that in the LC W56 and PBS groups (*p* < 0.01), and the stimulation index with 5 μg/mL of E2 protein in the pPG-E2-DCpep/LC W56 group was significantly higher compared to that in the pPG-E2/LC W56 group (*p* < 0.05), LC W56 group (*p* < 0.01), and PBS group (*p* < 0.01), showing a dose-dependent response. Moreover, significant levels (*p* < 0.01) of Th1-associated cytokine IFN-γ and Th2-associated cytokine IL-4 were produced in the culture supernatant of splenocytes obtained from the pPG-E2-DCpep/LC W56 group compared to that in the other groups (Figure 7D).

### 3.8. Challenge Experiment Results

**Figure 8:**
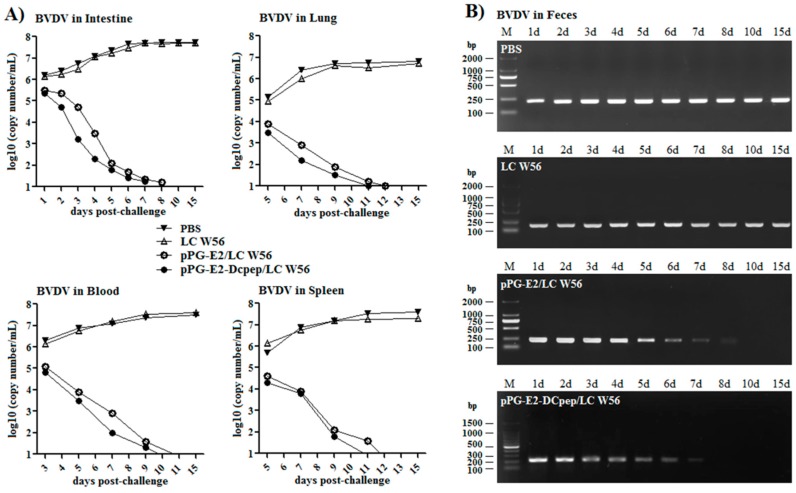
The elimination of the virus in the intestine, lung, blood, and spleen of the mice in PBS group, LC W56 group, pPG-E2/LC W56 group, and pPG-E2-DCpep/LC W56 group post-challenge with 10^5^ TCID_50_ BVDV (**A**) and the levels of virus excretion in feces of the mice in each group (**B**).

**Figure 9:**
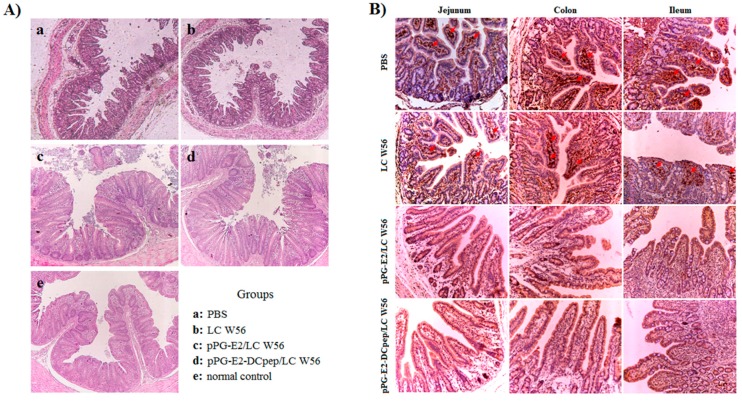
Histopathological changes (**A**) and immunohistochemistry (**B**) observed in the intestine of the mice from pPG-E2-DCpep/LC W56 group, pPG-E2/LC W56 group, LC W56 group, and PBS group on days 15 post-challenge with BVDV. Results showed that no abnormal histopathological changes were observed in mice orally immunized with the pPG-E2-DCpep/LC W56 and pPG-E2/LC W56, whereas prominent histopathological changes, including severe disruption of the intestinal structural integrity and shortening of the villi, developed in mice of LC W56 group and PBS group. Moreover, the immunohistochemical results of the intestine showed that large amounts of virus was observed in the jejunum, colon, and ileum of the mice in PBS group and LC W56 group on day 15 post-challenge, while no virus was detected in pPG-E2-DCpep/LC W56 group and pPG-E2/LC W56 group.

In this study, SPF BALB/c mice were used as animal model to evaluate the immune protection of the recombinant lactobacillus via oral vaccination. On day 42 after the primary immunization, the mice in each group were orally challenged with 200 μL containing 10^5^ TCID_50_ BVDV, and during 15-day challenge period, we observed that virus excretion in feces collected from the mice orally immunized with the strains pPG-E2-DCpep/LC W56 and pPG-E2/LC W56 gradually decreased as detected by reverse transcription PCR using primers BVDV-F/BVDV-R (as shown in Table 1). As shown in Figure 8B, little virus can be detected from the 7th day in the pPG-E2-DCpep/LC W56 group onwards post-challenge and from the 8th day in pPG-E2/LC W56 group onwards post-challenge, indicating efficient viral clearance. However, virus excretion in feces collected from the mice in the LC W56 group and PBS group still remained at a high level during the 15-day challenge period. At the same time, we detected the viral loads in the intestine, blood, lung, and spleen of the mice in each group post-challenge with BVDV by quantitive real-time RT-PCR, and results showed that the viral loads gradually decreased in these tissues of the immunized mice with pPG-E2-DCpep/LC W56 and pPG-E2/LC W56, while the viral loads in the mice that received either LC W56 or PBS gradually increased post-challenge (Figure 8A). Our results demonstrated that following the elimination of the virus in the intestine of the mice immunized with the probiotic vaccine, the virus in the internal circulation system could be effectively cleared. Moreover, after the challenge, the mice in each group were euthanized, and the intestine was excised for histopathological observation. As shown in Figure 9A, compared to that in the normal control group (e) (without being challenged), there were no abnormal histopathological changes observed in the intestine of the mice orally immunized with the strains pPG-E2-DCpep/LC W56 (d) and pPG-E2/LC W56 (c). However, there were obvious histopathological changes in the intestine of the mice in the LC W56 group (b) and PBS group (a), including disruption of the intestinal structural integrity and shortening of the villi. Subsequently, we detected the virus in the intestinal mucosa of the mice in each group by immunohistochemical assay with the HRP-conjugated mouse anti-E2 monoclonal antibody, and results showed that there were large amounts of virus observed in the jejunum, colon, and ileum of the mice in PBS group and LC W56 group on day 15 post-challenge, while no virus was detected in pPG-E2-DCpep/LC W56 group and pPG-E2/LC W56 group (Figure 9B).

## 4. Discussion

Bovine viral diarrhea virus (BVDV), an important viral pathogen of cattle, is responsible for significant losses to the cattle industry worldwide. Generally, in order to effectively control infectious diseases, the basic principles should include elimination of the pathogen reservoir and prevention or reduction of transmission from infected individuals to susceptible animals [34,35]. Currently, for the control of BVDV transmission, program designed to eradicate appear, which has been, or is being, adopted by several countries or regions [36,37,38]; however, the eradication of BVDV is still in its infancy, globally. Moreover, using vaccination to limit the spread of the disease associated with BVDV infection is important for cattle operations, and several BVDV vaccines, including modified live viral and inactivated viral vaccines, are available commercially. However, a major drawback of killed virus vaccines is the need to dose multiple times to achieve adequate antibody levels in the animals, which may also delay onset of immunity from the time of initial vaccination. In addition, the use of live attenuated vaccines is restricted in pregnant cattle due to safety concerns [35]. On the other hand, the invasion of BVDV often initiates at mucosal surfaces, such as respiratory and intestinal tract mucosa [17,39]. Therefore, vaccination inducing sIgA-based protective mucosal immunity via the mucosal approach could effectively prevent BVDV from entering the body via the mucosa and further spreading to the systemic circulation. Although, modified live viral and inactivated viral vaccines significantly contribute to reducing risks associated with BVDV infection, the vaccines administered by parenteral routes generally fail to induce effective anti-BVDV mucosal immune responses. Thus, the design of vaccines delivered via the mucosal route to induce anti-BVDV protective immunity could be an effective strategy. 

Lactobacillus strains make attractive candidates as antigen delivery carriers for the development of oral vaccines [40,41], since they are well known for probiotic effects on the health of humans and animals, can colonize the intestinal tracts [42,43], and exert their intrinsic immunoadjuvant activity [44,45]. In this study, a genetically engineered *Lactobacillus casei* expressing BVDV protective antigen E2 protein was generated using a constitutive expression vector pPG-T7g10 and its immunogenicity as an oral probiotic vaccine was evaluated using BALB/c mice as animal model. Currently, inducible expression systems, such as xylose [20,46] and nisin [47], are widely used to construct genetically engineered lactic acid bacteria (LAB) for vaccine development. However, the inducible system has many disadvantages. Particularly, the expression of antigen protein must be induced by a specific inducer, which limits the application of genetically engineered LAB in practice. Remarkably, the recombinant strain pPG-E2-DCpep/LC W56 constructed in this study, using the constitutive expression system that has been used in our previous studies [23,24,48], can express the protein of interest without the need of any inducers, exhibiting a significant advantage as compared with inducible expression systems.

For a live oral probiotic vaccine, colonization ability in the intestinal tracts is important and desirable. Thus, the colonization potentiality of the genetically engineered strain pPG-E2-DCpep/LC W56 labeled by the cFDA-SE probe was explored post-immunization, and results showed that the strain pPG-E2-DCpep/LC W56 can adhere and colonize in the intestinal tracts of mice, and the adhesion of the pPG-E2-DCpep/LC W56 was most prominent in the colon. On the other hand, the concentration of cFDA-SE probe used to label the recombinant strain would decrease with the increase of the levels of cFDA-SE-labeled pPG-E2-DCpep/LC W56. Therefore, although the detection rate of the cFDA-SE-labeled pPG-E2-DCpep/LC W56 gradually decreased each day, the amount of the pPG-E2-DCpep/LC W56 levels may still remain relatively high in the intestinal tracts. 

As an effective vaccine, it is important to elicit host’s immune responses from the time of initial vaccination rapidly. Dendritic cells, which act as antigen-presenting cells, are widely distributed beneath the gastrointestinal epithelium. Therefore, in order to improve the delivery efficiency of vaccine antigens to intestinal mucosa tissue for the quick and strong elicitation of host’s immune responses against infection, new generation of oral mucosal vaccines targeting dendritic cells has been suggested [23,25,26]. In this study, DCpep was introduced into the recombinant strain pPG-E2-DCpep/LC W56 followed by the analysis of its ability to promote intestinal mucosal DC activation in the Peyer’s patches (PPs) of the small intestine using flow cytometry assay. We observed that the ability of the strain pPG-E2-DCpep/LC W56 to promote intestinal mucosal DC activation was stronger than that of pPG-E2/LC W56 and pPG/LC W56. In addition, it is noteworthy that the non-engineered probiotics themselves could significantly promote DC activation compared to PBS control, indicating its immunoadjuvant activity.

As an effective oral vaccine, it is expected to be able to induce both intestinal mucosal and systemic immune responses efficiently. Thus, we evaluated the immunogenicity in mice orally administrated with strain pPG-E2-DCpep/LC W56. The sIgA antibody plays a key role in host protection against pathogenic infections via mucosa. We observed that a higher level of antigen-specific mucosal sIgA antibody, having anti-BVDV neutralizing activity, was produced in the intestinal mucosa of the mice orally immunized with the pPG-E2-DCpep/LC W56 from the 5th day onwards after the primary immunization compared to the other groups. Moreover, IgG antibody with anti-BVDV neutralizing activity in serum also contributes significantly to immune defense, by inhibiting viral spread to the systemic circulation. We observed that significant levels of antigen-specific IgG antibody was induced in serum of the mice orally immunized with the recombinant pPG-E2-DCpep/LC W56 from the 7th day onwards after the primary immunization compared to that in the other groups, and following the second booster immunization, a stronger anti-BVDV IgG response was detected in mice of pPG-E2-DCpep/LC W56 group, showing anti-BVDV neutralizing activity. Therefore, we demonstrated here that oral vaccination with strain pPG-E2-DCpep/LC W56 targeting DCs to deliver BVDV protective antigen E2 protein could strongly induce anti-BVDV mucosal and systemic immune responses. By contrast, although previous studies showed that submit vaccines and DNA vaccines developed with the BVDV E2 protein/gene could elicit effective immune responses via intramuscular injection [28,29,30,31], these vaccines could not induce specific sIgA-based immune response at mucosal surfaces to inhibit viral spread to the systemic circulation. Moreover, we observed that oral immunization with the strain pPG-E2-DCpep/LC W56 could promote the proliferation of CD4^+^ and CD8^+^ T lymphocytes more efficiently. At the same time, higher levels of Th1-associated cytokine IFN-γ and Th2-associated cytokine IL-4 were elicited in mice by the strain pPG-E2-DCpep/LC W56, indicating that pPG-E2-DCpep/LC W56 could also elicit cellular immune responses more efficiently.

Previous studies have demonstrated that mice can be used as animal model for the evaluation of experimental BVDV infection [49,50]. Therefore, in this study, we used the BALB/c mice as model to evaluate the immune protection of the strain pPG-E2-DCpep/LC W56 for the immunized mice against BVDV infection. The gastrointestinal clinical sign is one of parameters to define clinical protection to experimental BVDV infection [51,52]. We observed obvious histopathological changes, including disruption of the intestinal structural integrity and shortening of the villi, in the intestine of the mice which received the LC W56 and PBS after BVDV challenge, while there were no abnormal histopathological changes observed in the intestines of the mice orally immunized with the strains pPG-E2-DCpep/LC W56 and pPG-E2/LC W56. Further, the virus could be gradually cleared from the intestine of the mice orally immunized with the pPG-E2-DCpep/LC W56 and pPG-E2/LC W56 detected by immunohistochemistry assay and no virus was detected on day 15 post-challenge, but not in the mice received with PBS and LC W56. Here, our results clearly demonstrated that the probiotic vaccine could provide effective immune protection against BVDV infection via oral administration. Moreover, it is a real challenge for the oral probiotic vaccine to enter into the intestine through the rumen of ruminant, and our research is under way to further explore.

## 5. Conclusions

In conclusion, an oral probiotic vaccine against BVDV using *L. casei* W56 to deliver BVDV protective antigen E2 protein targeting intestinal DCs was developed in this study. Constitutive expression of the antigen protein of interest was identified by Western blotting and immunofluorescence assay. Using BALB/c mice as the animal model, the immunogenicity of the orally administered probiotic vaccine was evaluated, and the results showed that the probiotic vaccine could efficiently induce anti-BVDV mucosal, humoral, and cellular immune responses via oral vaccination, indicating a potential vaccination strategy against BVDV.

## Figures and Tables

**Table 1:** Primers used in this study.

Primers	Sequence (5′–3′)	Production Size
BVDV-E2-F	CGAGCTC^a^ATGCTCCCAGCCTGTAAACC	1122 bp
BVDV-E2-R	TTTGGGCCC^a^TTAACCTAAGGTCGTTTGTTCTGAT	
E2-DCpep	GGGCCC^a^ TGGACGTTGTGGAGTTGAATGATATGATGGATAAAA^b^*TGAGCCACCGCCACC*^c^TTAACCTAAGGTCGTTTGTTC	
BVDV-F	GGTAGCAACAGTGGTGAG	221 bp
BVDV-R	GTAGCAATACAGTGGGCC	

^a.^ Restriction enzyme sites used for cloning (underline); ^b.^ DCpep (in bold); ^c.^ Flexible amino acid (in italic).

## References

[1] Fray M.D., Paton D.J., Alenius S. (2000). The effects of bovine viral diarrhoea virus on cattle reproduction in relation to disease control. Anim. Reprod. Sci..

[2] Sarrazin S., Veldhuis A., Méroc E., Vangeel I., Laureyns J., Dewulf J., Caij A.B., Piepers S., Hooyberghs J., Ribbens S. (2013). Serological and virological BVDV prevalence and risk factor analysis for herds to be BVDV seropositive in Belgian cattle herds. Prev. Vet. Med..

[3] Szabára Á., Lang Z., Földi J., Hornyák Á., Abonyi T., Ózsvári L. (2016). Prevalence of bovine viral diarrhoea virus in cattle farms in hungary. Acta. Vet. Hung..

[4] Ma J.G., Cong W., Zhang F.H., Feng S.Y., Zhou D.H., Wang Y.M., Zhu X.Q., Yin H., Hu G.X. (2016). Seroprevalence and risk factors of bovine viral diarrhoea virus (BVDV) infection in yaks (*Bos grunniens*) in northwest China. Trop. Anim. Health Prod..

[5] Scharnböck B., Roch F.F., Richter V., Funke C., Firth C., Obritzhauser W., Baumgartner W., Käsbohrer A., Pinior B. (2018). A meta-analysis of bovine viral diarrhoea virus (BVDV) prevalences in the global cattle population. Sci. Rep..

[6] Neill J.D. (2013). Molecular biology of bovine viral diarrhea virus. Biologicals.

[7] Ridpath J.F., Bolin S.R., Dubovi E.J. (1994). Segregation of bovine viral diarrhea virus into genotypes. Virology.

[8] Giammarioli M., Ceglie L., Rossi E., Bazzucchi M., Casciari C., Petrini S., de Mia G.M. (2015). Increased genetic diversity of BVDV-1: Recent findings and implications thereof. Virus Genes.

[9] Decaro N., Lucente M.S., Lanave G., Gargano P., Larocca V., Losurdo M., Ciambrone L., Marino P.A., Parisi A., Casalinuovo F. (2017). Evidence for circulation of bovine viral diarrhoea virus type 2c in ruminants in southern Italy. Transbound. Emerg. Dis..

[10] Brodersen B.W. (2014). Bovine viral diarrhea virus infections: Manifestations of infection and recent advances in understanding pathogenesis and control. Vet. Pathol..

[11] Ståhl K., Alenius S. (2012). BVDV control and eradication in Europe-an update. JPN. J. Vet. Res..

[12] Lindberg A.L., Alenius S. (1999). Principles for eradication of bovine viral diarrhoea virus (BVDV) infections in cattle populations. Vet. Microbiol..

[13] Houe H., Lindberg A., Moennig V. (2006). Test strategies in bovine viral diarrhea virus control and eradication campaigns in Europe. J. Vet. Diagn. Investig..

[14] Lindberg A., Brownlie J., Gunn G.J., Houe H., Moennig V., Saatkamp H.W., Sandvik T., Valle P.S. (2006). The control of bovine viral diarrhoea virus in Europe: Today and in the future. Rev. Sci. Tech..

[15] Stott A.W., Humphry R.W., Gunn G.J., Higgins I., Hennessy T., O’Flaherty J., Graham D.A. (2012). Predicted costs and benefits of eradicating BVDV from Ireland. Irish Vet. J..

[16] Lanyon S., Reichel M. (2014). Bovine viral diarrhoea virus (‘pestivirus’) in Australia: To control or not to control?. Aust. Vet. J..

[17] Falcone E., Cordioli P., Tarantino M., Muscillo M., Sala G., Rosa G.L., Archetti I.L., Marianelli C., Lombardi G., Tollis M. (2003). Experimental infection of calves with bovine viral diarrhoea virus type-2 (bvdv-2) isolated from a contaminated vaccine. Vet. Res. Commun..

[18] Frink S., Grummer B., Pohlenz J.F., Liebler-Tenorio E.M. (2002). Changes in distribution and numbers of CD4+ and CD8+ T-lymphocytes in lymphoid tissues and intestinal mucosa in the early phase of experimentally induced early onset mucosal disease in cattle. J. Vet. Med. B Infect. Dis. Vet. Public Health.

[19] Xu Y., Li Y. (2008). Construction of recombinant *Lactobacillus casei* efficiently surface displayed and secreted porcine parvovirus VP2 protein and comparison of the immune responses induced by oral immunization. Immunology.

[20] Xu Y.G., Guan X.T., Liu Z.M., Tian C.Y., Cui L.C. (2015). Immunogenicity in swine of orally administered recombinant *Lactobacillus plantarum* expressing classical swine fever virus E2 protein in conjunction with thymosin α-1 as an adjuvant. Appl. Environ. Microbiol..

[21] Yu M., Qi R., Chen C., Yin J., Ma S., Shi W., Wu Y., Ge J., Jiang Y., Tang L. (2017). Immunogenicity of recombinant *Lactobacillus casei* expressing F4 (K88) fimbrial adhesin FaeG in conjunction with a heat-labile enterotoxin A (LTAK 63) and heat-labile enterotoxin B (LTB) of enterotoxigenic *Escherichia coli* as an oral adjuvant in mice. J. Appl. Microbiol..

[22] Yu M., Wang L., Ma S., Wang X., Wang Y., Xiao Y., Jiang Y., Qiao X., Tang L., Xu Y. (2017). Immunogenicity of eGFP-marked recombinant *Lactobacillus casei* against transmissible gastroenteritis virus and porcine epidemic diarrhea virus. Viruses.

[23] Wang X., Wang L., Huang X., Ma S., Yu M., Shi W., Qiao X., Tang L., Xu Y., Li Y. (2017). Oral delivery of probiotics expressing dendritic cell-targeting peptide fused with porcine epidemic diarrhea virus COE antigen: A promising vaccine strategy against PEDV. Viruses.

[24] Ma S., Wang L., Huang X., Wang X., Chen S., Shi W., Qiao X., Jiang Y., Tang L., Xu Y. (2018). Oral recombinant *Lactobacillus* vaccine targeting the intestinal microfold cells and dendritic cells for delivering the core neutralizing epitope of porcine epidemic diarrhea virus. Microb. Cell Fact..

[25] Wells J. (2011). Mucosal vaccination and therapy with genetically modified lactic acid bacteria. Annu. Rev. Food Sci. Technol..

[26] Owen J.L., Sahay B., Mohamadzadeh M. (2013). New generation of oral mucosal vaccines targeting dendritic cells. Curr. Opin. Chem. Biol..

[27] Mohamadzadeh M., Duong T., Sandwick S.J., Hoover T., Klaenhammer T.R. (2009). Dendritic cell targeting of *Bacillus anthracis* protective antigen expressed by *Lactobacillus acidophilus* protects mice from lethal challenge. Proc. Natl. Acad. Sci. USA.

[28] Bruschke C.J., Moormann R.J., van Oirschot J.T., van Rijn P.A. (1997). A subunit vaccine based on glycoprotein E2 of bovine virus diarrhea virus induces fetal protection in sheep against homologous challenge. Vaccine.

[29] Harpin S., Hurley D.J., Mbikay M., Talbot B., Elazhary Y. (1999). Vaccination of cattle with a DNA plasmid encoding the bovine viral diarrhoea virus major glycoprotein E2. J. Gen. Virol..

[30] Toth R.L., Nettleton P.F., McCrae M.A. (1999). Expression of the E2 envelope glycoprotein of bovine viral diarrhoea virus (BVDV) elicits virus-type specific neutralising antibodies. Vet. Microbiol..

[31] Nobiron I., Thompson I., Brownlie J., Collins M.E. (2001). Cytokine adjuvancy of BVDV DNA vaccine enhances both humoral and cellular immune responses in mice. Vaccine.

[32] Xu Y.X., Ayala A., Monfils B., Cioffi W.G., Chaudry I.H. (1997). Mechanism of intestinal mucosal immune dysfunction following trauma-hemorrhage: Increased apoptosis associated with elevated Fas expression in Peyer’s patches. J. Surg. Res..

[33] Fan J., Xie Y., Li X., Guo G., Meng Q., Xiu Y., Li T., Feng W., Ma L. (2009). The influence of Peyer’s patch apoptosis on intestinal mucosal immunity in burned mice. Burns.

[34] Walz P.H., Grooms D.L., Passler T., Ridpath J.F., Tremblay R., Step D.L., Callan R.J., Givens M.D. (2010). Control of bovine viral diarrhea virus in ruminants. J. Vet. Intern. Med..

[35] Newcomer B.W., Chamorro M.F., Walz P.H. (2017). Vaccination of cattle against bovine viral diarrhea virus. Vet. Microbiol..

[36] Presi P., Struchen R., Knight-Jones T., Scholl S., Heim D. (2011). Bovine viral diarrhea (BVD) eradication in Switzerland-Experiences of the first two years. Prev. Vet. Med..

[37] Graham D.A., Lynch M., Coughlan S., Doherty M.L., O’Neill R., Sammin D., O’Flaherty J. (2014). Development and review of the voluntary phase of a national BVD eradication programme in Ireland. Vet. Rec..

[38] Duncan A.J., Gunn G.J., Humphry R.W. (2016). Difficulties arising from the variety of testing schemes used for bovine viral diarrhoea virus (BVDV). Vet. Rec..

[39] Wang W., Shi X., Tong Q., Wu Y., Xia M.Q., Ji Y., Xue W., Wu H. (2014). A bovine viral diarrhea virus type 1a strain in China: Isolation, identification, and experimental infection in calves. Virol. J..

[40] Cano-Garrido O., Seras-Franzoso J., Garcia-Fruitós E. (2015). Lactic acid bacteria: Reviewing the potential of a promising delivery live vector for biomedical purposes. Microb. Cell Fact..

[41] Heiss S., Hörmann A., Tauer C., Sonnleitner M., Egger E., Grabherr R., Heinl S. (2016). Evaluation of novel inducible promoter/repressor systems for recombinant protein expression in *Lactobacillus plantarum*. Microb. Cell Fact..

[42] Alander M., Satokari R., Korpela R., Saxelin M., Vilpponen-Salmela T., Mattila-Sandholm T. (1999). Persistence of colonization of human colonic mucosa by a probiotic strain, *Lactobacillus rhamnosus* GG, after oral consumption. Appl. Environ. Microbiol..

[43] Xu Y., Li Y. (2007). Induction of immune responses in mice after intragastric administration of *Lactobacillus casei* producing porcine parvovirus VP2 protein. Appl. Environ. Microbiol..

[44] Ogawa T., Asai Y., Yasuda K., Sakamoto H. (2005). Oral immunoadjuvant activity of a newsymbiotic *Lactobacillus casei* subsp. casei in conjunction with dextran in BALB/cmice. Nutr. Res..

[45] Remus D.M., van Kranenburg R., van Swam I.I., Taverne N., Bongers R.S., Wels M., Wells J.M., Bron P.A., Kleerebezem M. (2012). Impact of 4 *Lactobacillus plantarum* capsular polysaccharide clusters on surface glycan composition and host cell signaling. Microb. Cell Fact..

[46] Zhao L., Guo Z., Liu J., Wang Z., Wang R., Li Y., Wang L., Xu Y., Tang L., Qiao X. (2017). Recombinant *Lactobacillus casei* expressing *Clostridium perfringens* toxoids α, β2, ε and β1 gives protection against *Clostridium perfringens* in rabbits. Vaccine.

[47] Hoang V.V., Ochi T., Kurata K., Arita Y., Ogasahara Y., Enomoto K. (2018). Nisin-induced expression of recombinant T cell epitopes of major Japanese cedar pollen allergens in *Lactococcus lactis*. Appl. Microbiol. Biotechnol..

[48] Gao X., Ma Y., Wang Z., Bai J., Jia S., Feng B., Jiang Y., Cui W., Tang L., Li Y. (2019). Oral immunization of mice with a probiotic *Lactobacillus casei* constitutively expressing the α-toxoid induces protective immunity against *Clostridium perfringens* α-toxin. Virulence.

[49] Seong G., Oem J.K., Lee K.H., Choi K.S. (2015). Experimental infection of mice with bovine viral diarrhea virus. Arch. Virol..

[50] Lee K.H., Han D.G., Kim S., Choi E.J., Choi K.S. (2018). Experimental infection of mice with noncytopathic bovine viral diarrhea virus 2 increases the number of megakaryocytes in bone marrow. Virol. J..

[51] Xue W., Mattick D., Smith L., Umbaugh J., Trigo E. (2010). Vaccination with a modified-live bovine viral diarrhea virus (BVDV) type 1a vaccine completely protected calves against challenge with BVDV type 1b strains. Vaccine.

[52] Palomares R.A., Givens M.D., Wright J.C., Walz P.H., Brock K.V. (2012). Valuation of the onset of protection induced by a modified-live virus vaccine in calves challenge inoculated with type 1b bovine viral diarrhea virus. Am. J. Vet. Res..

